# A gene expression programme induced by bovine colostrum whey promotes growth and wound-healing processes in intestinal epithelial cells

**DOI:** 10.1017/jns.2014.56

**Published:** 2014-11-13

**Authors:** M. Blais, Y. Pouliot, S. Gauthier, Y. Boutin, M. Lessard

**Affiliations:** 1 Dairy and Swine R & D Centre, Sherbrooke, QC, Canada; 2 INAF, Université Laval, Québec, Canada; 3 TransBIOTech, Lévis, QC, Canada

**Keywords:** Bovine colostrum whey, Intestinal epithelial cells, Wound healing, Gene expression, EGF, epidermal growth factor, EMT, epithelial–mesenchymal transition, FBS, fetal bovine serum, qPCR, quantitative PCR, TGF-β, transforming growth factor-beta

## Abstract

Bovine colostrum is well known for its beneficial properties on health and development. It contains a wide variety of bioactive ingredients that are known to promote a number of cellular processes. Therefore the use of colostrum whey as a feed additive to promote intestinal health has been proposed, yet little is known about mechanisms implicated in its beneficial properties on intestinal epithelial cells. In the present paper, casein were removed from bovine colostrum and the remaining liquid, rich in bioactive compounds, was evaluated for its capacity to modulate cellular processes in porcine intestinal epithelial cell line IPEC-J2 and human colon adenocarcinoma cell line Caco-2/15. First, we verified the effect of colostrum whey and cheese whey on processes involved in intestinal wound healing, including cell proliferation, attachment, morphology and migration. Our results showed that colostrum whey promoted proliferation and migration, and decreased specifically the attachment of Caco-2/15 cells on the culture dish. On the other hand, cheese whey induced proliferation and morphological changes in IPEC-J2 cells, but failed to induce migration. The gene expression profile of IPEC-J2 cells following colostrum whey treatment was evaluated by microarray analysis. Results revealed that the expression of a significant number of genes involved in cell migration, adhesion and proliferation was indeed affected in colostrum whey-treated cells. In conclusion, colostrum specific bioactive content could be beneficial for intestinal epithelial cell homoeostasis by controlling biological processes implicated in wound healing through a precise gene expression programme.

Intestinal epithelium integrity is crucial to maintain barrier function of the gastrointestinal tract. For numbers of digestive disorders, including inflammatory bowel diseases, the disruption of barrier integrity is a key event determining the outcomes of the disease^(^^1^^,^^2^^)^. To restore tissue homoeostasis following injury, a rapid activation of the wound-healing process is required. First, wound healing is initiated by epithelial restitution, during which cells surrounding the wound lose their polarity, undergo complex alterations of their adhesion properties, change their morphology and migrate toward the denuded area to cover the defect^(^^3^^–^^5^^)^. This is followed by cell proliferation, which is triggered to fill the wound with new enterocytes. Eventually, cell differentiation occurs to re-establish tissue architecture and function.

There is a wide variety of factors that can affect intestinal restitution and proliferation, including growth factors, such as transforming growth factor-beta (TGF-β) and epidermal growth factor (EGF), cytokines, regulatory peptides and SCFA^(^^3^^,^^6^^)^. Although many of these molecules have been studied and characterised extensively and their effect on intestinal epithelial cells have been described, little is known about the outcomes of their combination and the importance of their dosage. Interestingly, the content of colostrum, the first milk produced following birth, is rich in these compounds. In fact, in human subjects as well as in numerous animals, colostrum consumption after birth triggers a significant increase of intestinal size and changes in the tissue structure, positively affecting gastrointestinal functions^(^^7^^)^. A decrease in Ig and growth factors is observed within the first milkings^(^^8^^,^^9^^)^, when colostrum production is replaced by milk.

Dairy calf management practices have evolved considerably over the past 100 years, leading to considerable increase of colostrum production, often exceeding the calf's needs^(^^10^^,^^11^^)^. Consequently, there is growing interest to exploit this dairy product for medical and nutritional applications. Since most of bioactive components present in colostrum and milk are found in the whey fraction following casein removal^(^^12^^,^^13^^)^, the use of colostrum whey to promote intestinal health of human subjects and animals has been proposed, but better understanding of mechanisms involved in its beneficial properties on intestinal epithelial cells is required.

In the present study, we investigated the effect of colostrum whey, a complex natural source of bioactive ingredients, on mechanisms involved in the wound-healing process using porcine IPEC-J2 and human Caco-2/15 cells. IPEC-J2 cells were selected for their great advantages compared with other intestinal epithelial cell lines, including the facts that they are non-transformed and they have the potential to differentiate and form polarised monolayers^(^^14^^)^. Moreover, the swine model has been increasingly suggested over the past few years as a suitable model for human^(^^15^^–^^17^^)^. First, we verified the effect of colostrum whey and cheese whey on cell proliferation, adhesion, morphology and migration in IPEC-J2 and Caco-2/15 cells. The effect of colostrum whey was further investigated in IPEC-J2 cells, by evaluating gene expression profile following colostrum whey treatment using microarray analysis.

## Materials and methods

### Cell culture

Non-transformed porcine intestinal epithelial IPEC-J2 cells, derived from newborn piglet jejunum^(^^18^^)^, were a kind gift from Dr Joshua Gong (Agriculture and Agri-Food Canada, Guelph, Ontario, Canada). Cells were cultured in Dulbecco's modified Eagle's medium/Ham's F-12 [1:1] (Wisent) supplemented with 5 % heat-inactivated fetal bovine serum (FBS; Wisent), 1 % insulin–transferrin–selenium (premix; BD), 1 % glutamine and 5 ng/ml EGF (Wisent). For the induction of differentiation, confluent IPEC-J2 cells were grown in the FBS-deprived medium supplemented with 10^−7^ m dexamethasone (Sigma-Aldrich Canada) for 10 d. The Caco-2/15 human colon adenocarcinoma cell line^(^^19^^)^ was cultured in Dulbecco's modified Eagle's medium high glucose (Invitrogen) supplemented with 10 % FBS, 25 mm HEPES and glutamax (Invitrogen).

### Milk fractions

Bovine colostrum samples from the second and third milkings were collected from Holstein cows at the Laval University Animal Sciences Research Center (Deschambault, QC, Canada). As described by Montoni *et al.*^(^^9^^)^, colostrum samples (total lacteal secretion) were collected within 24 h from fourteen Holstein cows aged 23–110 months (primapara or 2–7-calf multipara heifers). They were immediately frozen and kept at −18°C. These samples were used to prepare colostrum whey, as described previously^(^^9^^)^. Briefly, colostrum samples were thawed, centrifuged at 10 000 ***g*** for 30 min at 4°C (Sorvall model RC-5B, GSA rotor, DuPont Instruments) and the solid fat layer was then carefully removed manually. The samples were then acidified to pH 4·6 with 1 m-HCl, and caseins were removed by centrifugation at 12 000 ***g*** for 15 min at 4°C. The crude whey was collected and the pH was adjusted to 7·0 with 1 m-NaOH. The colostrum whey samples were freeze-dried using a RePP model FFD-42-WS (The Virtis Co. Inc.). Fresh Mozzarella cheese whey obtained from a local cheese factory (L'Ancêtre) was skimmed by using a pilot-scale milk separator (Alfa Laval). Bacterial contamination of cheese whey was reduced by microfiltration (TetraPak MSF1) through a 1·4 µm membrane (Membralox™). Microfiltered whey was concentrated by ultrafiltration (UF) through a 5 kDa membrane (Romicon, Koch Membrane Systems), freeze dried and stored at −20°C. Final protein concentrations for colostrum whey and cheese whey were 68·073 and 71·815 %, respectively. Both whey products were irradiated with a dose of 5 kGy using a Gammacell 220 irradiator unit (Atomic Energy of Canada Ltd) and refrozen at −20°C. For the experiments, milk fractions were diluted in OptiMEM (Invitrogen).

### Proliferation assay

IPEC-J2 and Caco-2/15 cells were seeded at a density of 10^4^ cells/well in a ninety-six-well plate and allowed to adhere to the plate overnight. Cells were further incubated for 24 h with increasing doses of milk fractions (0, 0·1, 1 and 10 mg/ml) in the serum-deprived culture medium. Cell proliferation was measured using the 2,3-bis-(2-methoxy-4-nitro-5-sulfophenyl)-2H-tetrazolium-5-carboxanilide (XTT; InVitrogen), which assess cell viability and proliferation as a function of redox potential. Briefly, fresh 2,3-bis-(2-methoxy-4-nitro-5-sulfophenyl)-2H-tetrazolium-5-carboxanilide stock solution (1 mg/ml in PBS) was prepared, and PMS (phenazine methosulfate 15 mg/ml in PBS, store in dark and −20°C) was diluted 1:100 in PBS. PMS was added to 2,3-bis-(2-methoxy-4-nitro-5-sulfophenyl)-2H-tetrazolium-5-carboxanilide solution (40:1), and 50 µl of the mix was added to each wells. Cells were incubated for 1 h before the absorbance was measured at 450/630 nm. The absorbance in untreated cells (0·0 mg/ml) was set as 100 %. In total, four independent experiments were done in triplicate.

### Attachment studies

Immediately after trypsinisation, IPEC-J2 and Caco-2/15 cells were washed twice in the serum-free medium by centrifugation, and resuspended in their respective growing media supplemented with FBS, with or without colostrum whey (10 mg/ml) or cheese whey (10 mg/ml). Cells were seeded at the density of 10^5^/ml in 100-mm cell culture dishes. Attachment to the culture dish was measured after 18 h. For cell count, unattached cells were washed 3 times with PBS, detached using trypsin, resuspended in 10 ml of Dulbecco's modified Eagle's medium-5 % FBS and counted using a ‘Countess™ Automated Cell Counter’ (Invitrogen). Cells from three independent experiments were counted in triplicate.

### Cell morphology

For cell morphology observations, IPEC-J2 and Caco-2/15 cells were resuspended in their respective growing media supplemented with FBS, with increasing concentrations (0·0, 0·1, 1 and 10 mg/ml) of colostrum or cheese wheys. Cells were seeded at the density of 10^5^/ml in 100-mm cell culture dishes. Morphology was observed after 48 h. Cells were viewed with the Primo Vert microscope and photographed using the digital image processing software Axiovision 4.8 (Zeiss, Toronto, Ontario, Canada). Images are representative of three independent experiments.

### Wound-healing assay

IPEC-J2 cells were seeded at the density of 10^5^ cells/ml in 100-mm cell culture dishes. After reaching confluence, cells were allowed to differentiate for 10 d. In every culture dishes, three scratches of 10 mm were made on cell monolayers with a sterile razor blade. Detached cells were rinsed off three times with the serum-free Dulbecco's modified Eagle's medium. Milk fractions (10 mg/ml) were added to cells in the culture medium deprived of FBS, insulin–transferrin–selenium and EGF. Cells were photographed before and 18 h after wounding, using a Primo Vert microscope (Zeiss). Cell migration was quantified with the digital image-processing software Axiovision 4.8 (Zeiss) by measuring the surface covered by migrating cells, starting at the edge of each scratch. Images are representative of three independent experiments.

### Microarray analysis

Total RNAs from differentiated IPEC-J2 cells treated without (control) or with colostrum whey (10 mg/ml) for 2 h were extracted using Qiagen RNeasy kit (Qiagen). RNA sample quality was determined with the Agilent 2100 Bioanalyzer (Agilent Technology), using the RNA 6000 Nano Kit. RIN measured were higher than 9 for all samples. cDNA synthesis was performed with 25 ng of RNA and hybridisation with a Agilent Porcine Gene Expression Microarray (catalogue #G2519F-026440) at the Microarray platform of the McGill University and Genome Quebec Innovation Centre. The microarray contains 43 603 probes, with a third of these probes representing well characterised genes. Three independent experiments were done for each condition. Data analysis, normalisation, average difference and expression for each feature on the microarray were done using the Flexarray software version 1.6.1.

### Functional classification

Genes with expression levels increased or decreased more than 2-fold (−1 > fold change (log2) >1, *P* < 0·05) were selected and analysed according to their Gene Ontology classification, using Toppgene Suite (Transcriptome, ontology, phenotype, proteome and pharmacome annotations based gene list, http://toppgene.cchmc.org/). To identify biological processes significantly affected in colostrum-treated cells, Bonferroni correction was used with a *P*-value cut-off set at 0·05.

### Quantitative PCR analysis

Differentiated IPEC-J2 cells were treated with colostrum whey (10 mg/ml) or left untreated for 2 h. RNAs were prepared with Qiagen RNeasy kit (Qiagen). cDNAs were synthesised using oligo(dT) and Superscript II reverse transcriptase (Invitrogen), following the manufacturer's protocol. Quantitative PCR (qPCR) was performed by using Power SYBR Green PCR Master Mix and the 7500 fast real-time PCR system (Applied Biosystems, Life Technologies Inc). Primers designed with Primer-Blast (http://www.ncbi.nlm.nih.gov/tools/primer-blast) are listed in Table 1. All data were analysed with the ∆∆CT method, using β-actin and PPIA as internal controls.

**Table 1.** List of primers for quantitative PCR analysis of colostrum-regulated gene targets identified by microarray analysis

Gene symbol	RefSeqAccession	Primer	Sequence (5′– > 3′)	Start	Stop
AREG	NM_214376	Forward	CACAGCTCCGCGGGACCAAT	172	191
		Reverse	CCGTTGACGTCCAGTCCAGCA	281	261
CDKN2B	NM_214124	Forward	GCAAGTGGAGACCGTGCGTCA	82	102
		Reverse	CACGTGCGCTGCCCATCATCA	188	168
CXCR4	NM_213773	Forward	AAGGATGGACGCGCCACACA	23	42
		Reverse	GCATCCGAGAAGCGTGCCCC	123	104
F3	NM_213785	Forward	AAGTCGCATTGAGTGCACCAGCC	818	840
		Reverse	ACACAGGCGGGACGAGGACG	927	908
HAS3	NM_001001268	Forward	GCCGGCTTCTTTGTGTGGCG	436	455
		Reverse	TGCTGGCCCGCACCACATTC	544	525
HBEGF	NM_214299	Forward	CGTCGGTGGTGCTGAAGCTCT	14	34
		Reverse	ATTGCTGGTTCCATCCGCCAGC	117	96
IL-8	NM_213867	Forward	CAGCCCGTGTCAACATGACTTCCA	70	93
		Reverse	GCACTGGCATCGAAGTTCTGCAC	191	169
NGF	XM_001929549	Forward	CGAGAGGGAGAGGGTGGCTGG	18	38
		Reverse	AACGCCCACCTGCTTGTTGC	117	98
PLAU	NM_213945	Forward	GGTGAGAGTCACCGGCCTGC	570	589
		Reverse	TAAAGCGGGGCCTCAGAGCCT	669	649
PTGS1	XM_001926129	Forward	GCACCGGTGAACCCCTGTTGT	126	146
		Reverse	CAGAGTAGCCCGTGCGGGTG	231	212
SERPINE1	NM_213910	Forward	CCACCCCCGACGGCCATTAC	847	866
		Reverse	TGGTGAGGGCGGAGAGAGGC	960	941
SMAD7	NM_001244175	Forward	GATGCTGTGCCTTCCTCCGCT	676	696
		Reverse	GTGACCGATCCCCAGGCTCCA	775	755
TAGAP	NM_001243543	Forward	CGGCACATTCCAGCGTTGCC	1014	1033
		Reverse	ACGTCGGGGTCGGGATCGTT	1125	1106
β-actin	XM_003357928	Forward	CTCTTCCAGCCCTCCTTCCT	447	466
		Reverse	GCGTAGAGGTCCTTCCTGATGT	518	497
PPIA	NM_214353	Forward	TGCAGACAAAGTTCCAAAGACAG	75	97
		Reverse	GCCACCAGTGCCATTATGG	225	207

### Statistical methods

Comparisons of quantitative data were analysed by a one-way ANOVA. When a significant difference was identified by ANOVA (*P* < 0·05), Dunnett's adjustment (*post hoc*) was used to identify differences between treatments and the control untreated cells (*P* < 0·05). Analysis was performed using the SigmaPlot software (version 12.5). All data were found to comply with ANOVA assumptions. For microarray and qPCR results, fold change between colostrum whey-treated groups and untreated control groups were analysed in log2 scale, and comparisons of data were done using Welch's *t*-tests. Data are considered significant when *P* < 0·05. Data are reported as means with their standard errors.

## Results

**Fig. 1.**
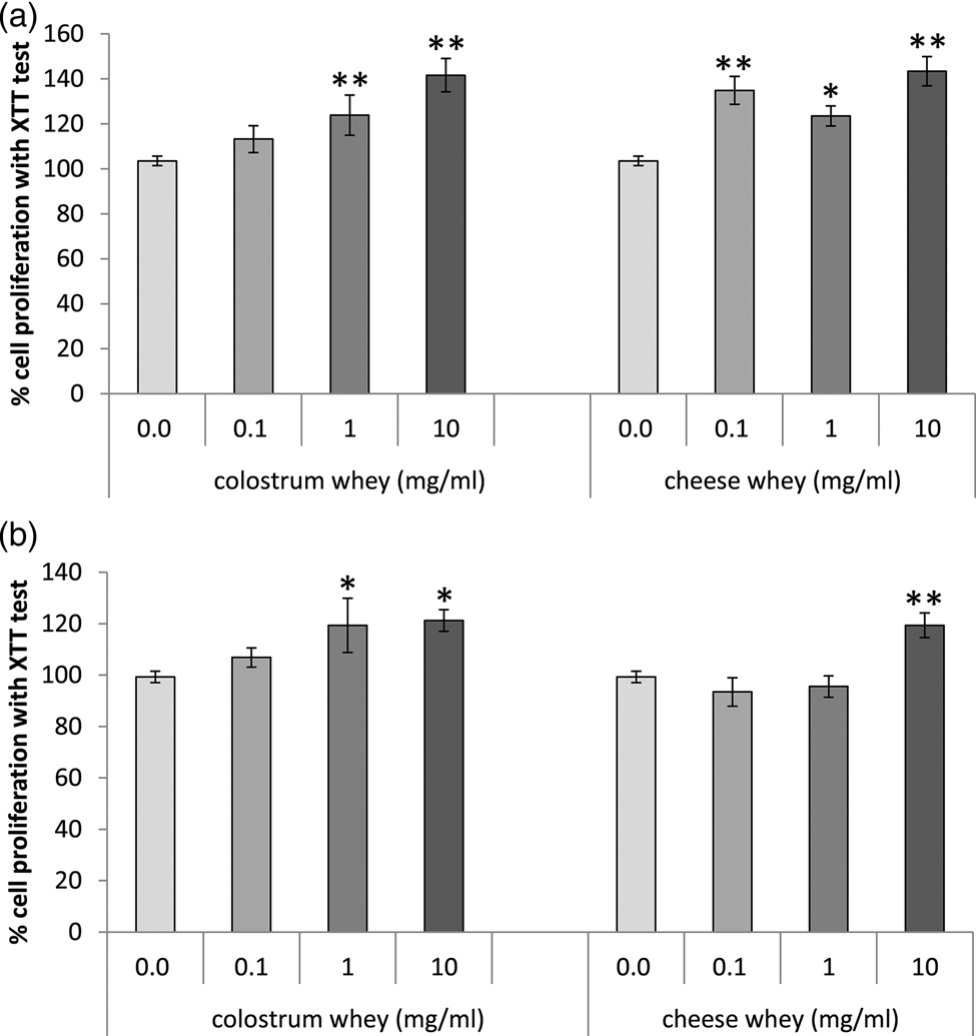
Colostrum and cheese whey treatments increased cell proliferation in intestinal epithelial cells. (a) IPEC-J2 and (b) Caco-2/15 cells were incubated with different concentrations of colostrum whey or cheese whey for 24 h. Cell proliferation was measured with an 2,3-bis-(2-methoxy-4-nitro-5-sulfophenyl)-2H-tetrazolium-5-carboxanilide (XTT) assay. Values are the mean percentage of proliferating cells compared with control cells (0 mg/ml), as determined by the XTT test, with means with their standard errors of four independent experiments done in triplicate. Mean values were significantly different from control by the *post hoc* analysis. **P < 0·01; *P < 0·05.

The impact of colostrum whey on biological processes involved in wound healing were evaluated in intestinal epithelial cells and compared with effects mediated by cheese whey. First 2,3-bis-(2-methoxy-4-nitro-5-sulfophenyl)-2H-tetrazolium-5-carboxanilide assay was performed in both porcine IPEC-J2 and human Caco-2/15 cells in order to determine the effect of wheys on cell proliferation, and different concentrations were tested to identify the dose to use in further experiments. Results showed that both colostrum and cheese wheys increased cell proliferation, with the highest values observed with the highest concentration tested (10 mg/ml; Fig. 1). Therefore, the highest dose was selected for each whey to test cell attachment, cell migration and to measure gene expression.

**Fig. 2.**
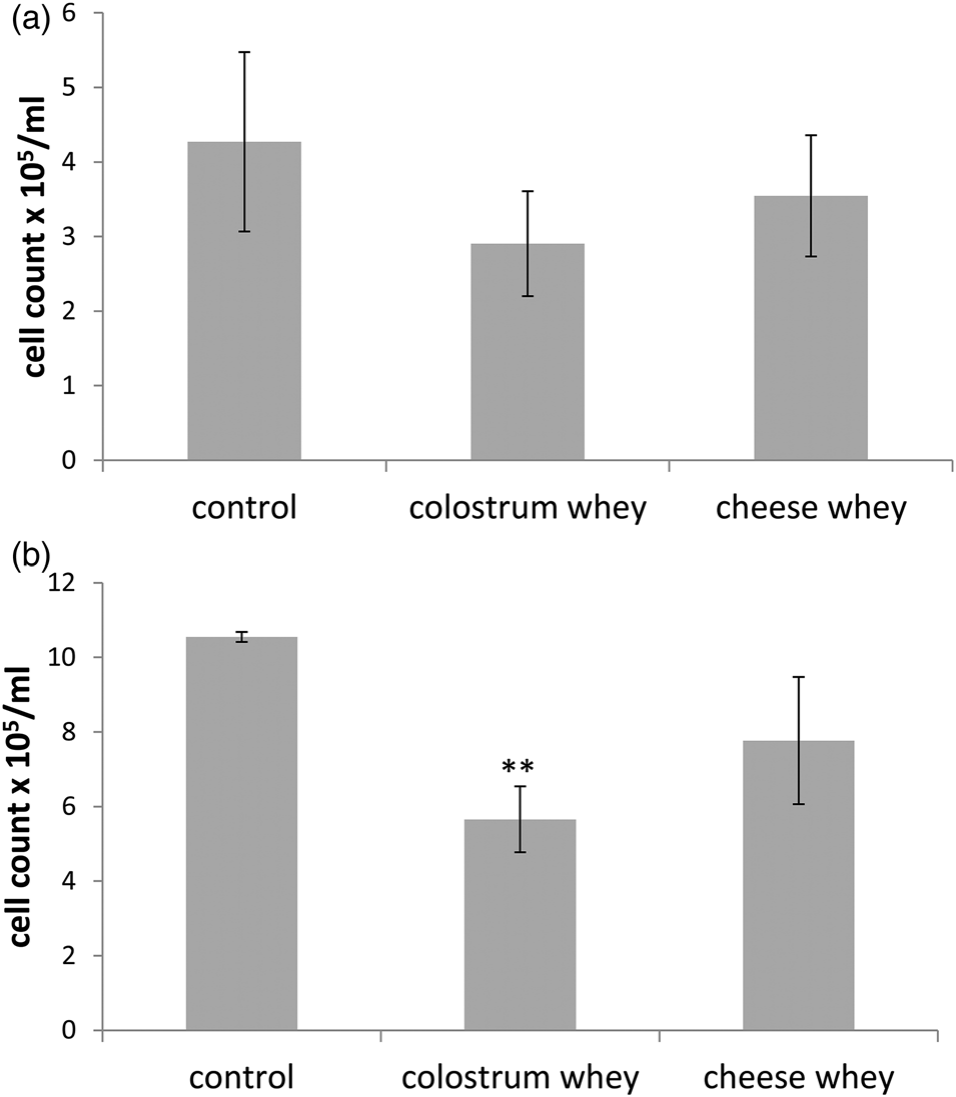
Cell attachment is affected specifically in Caco-2/15 cells treated with colostrum whey. (a) IPEC-J2 and (b) Caco-2/15 cells were recovered after trypsinisation and resuspended in the FBS-supplemented medium, with or without colostrum whey (10 mg/ml) or cheese whey (10 mg/ml). Cells were seeded and allowed to attach to the cell culture dish for 18 h, followed by cell count. Values are the mean of viable cells/ml, with means with their standard errors of three independent experiments. Mean values were significantly different from control by the *post hoc* analysis. **P < 0·01.

Cell adhesion process was evaluated by measuring IPEC-J2 and Caco-2 cells attachment to the culture dish. The quantification of attached cells revealed that neither colostrum whey nor cheese whey affected cell capacity to attach to the culture dish in IPEC-J2 cells, while colostrum whey treatment significantly decreased (*P* < 0·01) the attachment of Caco-2/15 cells (Fig. 2(B)).

**Fig. 3.**
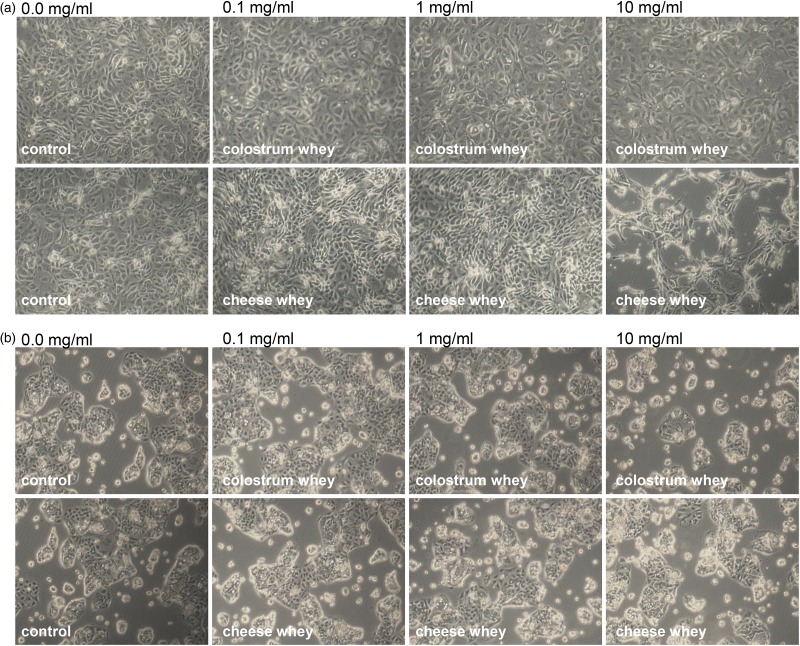
Morphological changes are observed in newly attached IPEC-J2 cell treated with colostrum and cheese wheys. (a) IPEC-J2 and (b) Caco-2/15 cells were recovered after trypsinisation and resuspended in Dulbecco's modified Eagle's medium supplemented with increasing concentrations (0·0, 0·1, 1 and 10 mg/ml) of colostrum or cheese wheys. Cells were seeded in cell culture dishes, and morphology was observed by microscopy after 48 h of incubation. Images shown are representative of three independent experiments.

Interestingly, morphological changes were noticed in IPEC-J2 cells following the attachment assay. These morphological changes were further investigated by allowing cells to attach to the culture dish for 48 h following resuspension, with or without decreasing concentrations of colostrum or cheese wheys. While important morphological changes were confirmed in newly attached IPEC-J2 cells following cheese whey treatment, changes were less significant in colostrum whey-treated cells (Fig. 3). Cells were stretched out with branched cytoplasm, and were more disjointed than control cells. Morphological changes subsided with lower concentrations of wheys. In Caco-2/15 cells, no morphological changes were observed, while a decrease of attached cells was noticeable in colostrum whey (10 mg/ml)-treated cells, confirming results obtained with the attachment assay.

**Fig. 4.**
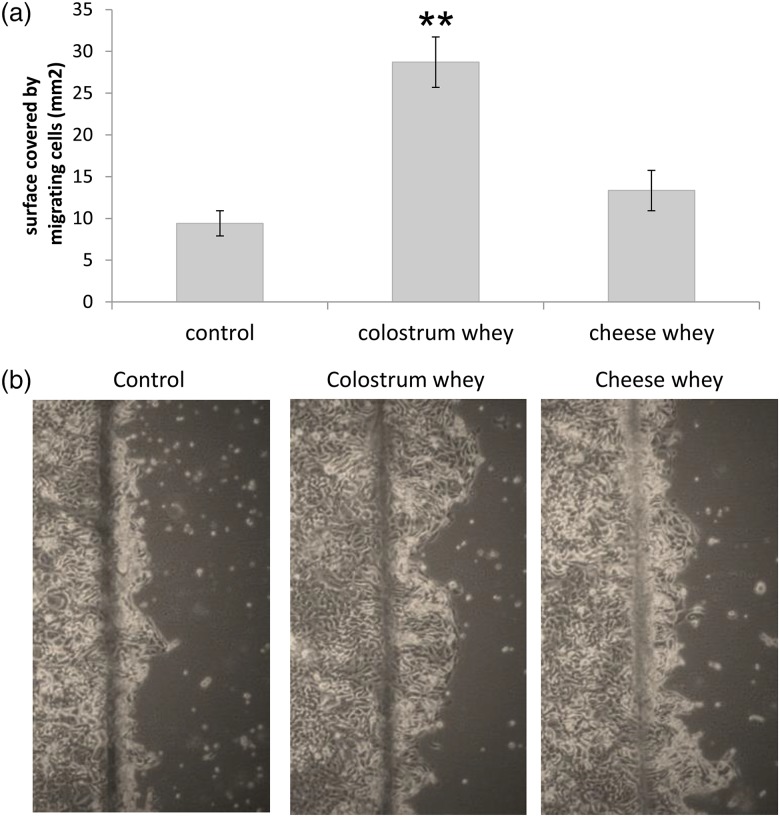
Colostrum whey promotes IPEC-J2 cell migration. IPEC-J2 monolayers were wounded with a razor blade, before treatment with colostrum whey or cheese whey (10 mg/ml). Migration was (a) measured and (b) photographed after 18 h. The graph represents mean values from three independent experiments with their standard errors. Mean values were significantly different from control by *post hoc* analysis. **P < 0·01.

Finally, effect of colostrum and cheese wheys on cell migration was assessed by wound healing assay using non-proliferative differentiated IPEC-J2 cells. Results showed a significant increase in IPEC-J2 cell migration in colostrum whey-treated cells compared with untreated cells, while cheese whey had no impact on cell migration (Fig. 4). No migration was observed in differentiated Caco-2/15 cells (data not shown).

To further establish the mechanisms involved in the regulation of cellular processes by colostrum whey, a gene expression profile of colostrum-treated and -untreated IPEC-J2 cells was obtained by microarray analysis. Genes that showed significant 2-fold increase or decrease expression (−1> log2-fold change >1; *P* < 0·05) were selected. In total, 102 probes were significantly increased or decreased more than 2-fold in colostrum whey-treated cells, in which twenty-nine known and characterised genes were identified. These genes are listed in Table 2. Also, thirteen genes were selected from the microarray results (eleven genes involved in proliferation, migration, or adhesion, as well as two genes involved in other biological processes), and their expression in IPEC-J2 cells treated with colostrum whey was verified by qPCR. Results obtained with qPCR are shown in the right panel of Table 2, and confirmed the increase of CDKN2B, NGF, SMAD7 and TAGAP (log2-fold change >1), as well as the increase of AREG, CXCR4, F3, HAS3, IL-8 and PLAU (log2-fold change >0·5).

**Table 2.** List of known genes significantly increased or decreased more than 2-fold in colostrum whey-treated IPEC-J2 cells, as determined by microarray analysis and quantitative PCR analysis

				Fold change (log2)	
Gene symbol	Gene name	Primary accession	Probe name	Microarray	q-PCR
AREG	Amphiregulin	NM_214376	A_72_P440121	1·18*	0·75†
			A_72_P302799	1*	
CDKN2B	Cyclin-dependent kinase inhibitor 2B	NM_214124	A_72_P077616	1·44*	1·49*
CITED2	Cbp/p300-interacting transactivator 2-like	XM_001925774	A_72_P491235	−1·27*	
			A_72_P555835	−1·07	
			A_72_P548306	−1·07*	
			A_72_P192006	−1·17*	
			A_72_P500550	−1·23*	
CSF2	Colony-stimulating factor 2	NM_214118	A_72_P077726	2·12*	
CXCR4	Chemokine (C–X–C motif) receptor 4	AK238923	A_72_P661857	1·02*	0·98†
CYP1A1	Cytochrome P450 1A1	NM_214412	A_72_P443804	1·73*	
			A_72_P223362	1·6*	
CYR61	Cysteine-rich, angiogenic inducer, 61	ENSSSCT00000007604	A_72_P580852	1·28*	
			A_72_P688281	1·23*	
			A_72_P552021	1·1*	
			A_72_P319303	1*	
F3	Tissue factor	NM_213785	A_72_P177461	1·45*	0·71*
			A_72_P641835	1·37*	
FOS	FBJ murine osteosarcoma viral oncogene homologue	NM_001123113	A_72_P441284	1·12*	
			A_72_P670145	1·11*	
			A_72_P205307	1·04*	
GADD45A	Growth arrest and DNA-damage-inducible, alpha	NM_001044599	A_72_P146986	1·41*	
GADD45G	Growth arrest and DNA-damage-inducible, gamma	ENSSSCT00000010506	A_72_P011276	1·05*	
HAS3	Hyaluronan synthase 3	NM_001001268	A_72_P303284	1·36*	0·86*
HBEGF	Heparin-binding EGF-like growth factor	NM_214299	A_72_P035756	1·27*	1·44
			A_72_P178751	1·06*	
HR	Hairless protein	TC543384	A_72_P355953	2·59*	
IL-8	IL-8	NM_213867	A_72_P232367	1·04*	0·69†
NGF	Beta-nerve growth factor-like	ENSSSCT00000007392	A_72_P290809	1·54*	1·30*
PLAU	Plasminogen activator, urokinase	NM_213945	A_72_P010246	1·46*	0·97*
			A_72_P073101	1·02*	
PTGS1	Prostaglandin-endoperoxide synthase 1	AK232305	A_72_P063591	1·04*	0·80
RNF39	RING finger protein 39-like	XM_003128244	A_72_P108096	1·89*	
SERPINE1	Serpin peptidase inhibitor, clade E, member 1	NM_213910	A_72_P077351	1·33*	0·75
			A_72_P585596	1·14*	
			A_72_P668499	1·12*	
SLITRK6	SLIT and NTRK-like family, member 6	ENSSSCT00000010401	A_72_P452998	−1·01*	
SMAD7	SMAD family member 7	ENSSSCT00000004979	A_72_P492369	1·35*	3·16*
SOX9	SRY (sex-determining region Y)-box 9	NM_213843	A_72_P146931	1·08*	
SPB9	Serine protease inhibitor 9	TC587447	A_72_P334903	1·3*	
TAGAP	t-cell activation Rho GTPase-activating protein	ENSSSCT00000004481	A_72_P709805	1·75*	1·24*
TIPARP	TCDD-inducible poly(ADP-ribose) polymerase	AK343870	A_72_P024051	1·37*	
			A_72_P036711	1·18*	
WNT11	Wingless-type MMTV integration site family, 11	ENSSSCT00000016217	A_72_P219917	1·37*	
XKR8	XK-related protein 8-like	XM_003127736	A_72_P259637	1·24*	
ZNF503	Zinc finger protein 503-like	XM_003133082	A_72_P161271	−1·11*	

Fold changes = log2 (colostrum whey/control). *P < 0·05, †*P* < 0·1.

**Fig. 5.**
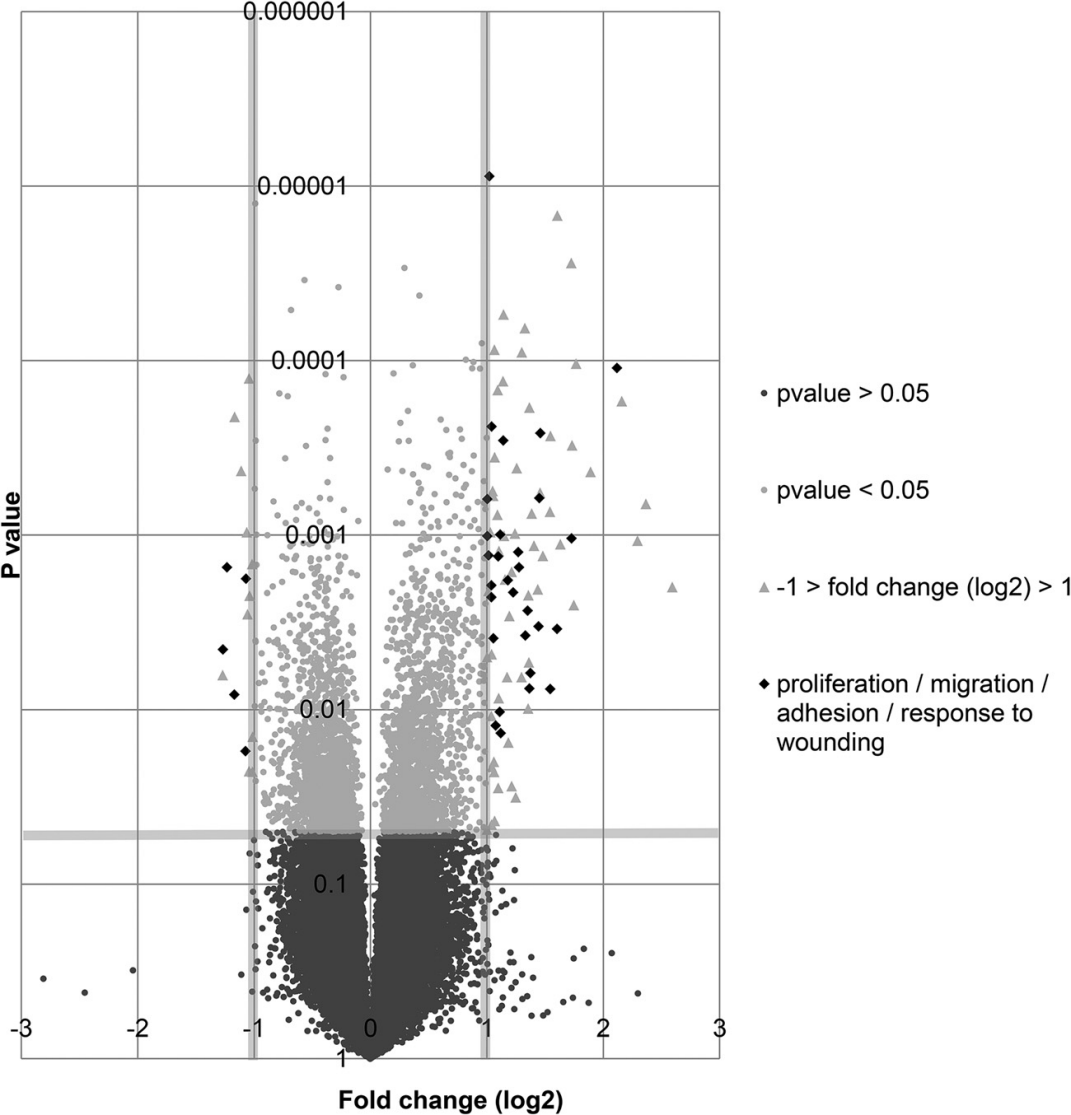
Colostrum whey treatment significantly increased or decreased gene expression involved in proliferation, adhesion or migration of IPEC-J2. Gene expression in IPEC-J2 cells treated with colostrum whey (10 mg/ml) for 2 h, as determined by microarray analysis, is illustrated in the volcano plot. Genes involved in proliferation, adhesion or cell migration are represented in diamond-shape.

To classify up-regulated and down-regulated genes according to their biological processes, ToppGene suite database for functional enrichment based on Ontologies (Gene Ontology, Pathway) was used. This analysis revealed that, indeed, biological processes involved in wound healing were significantly affected in colostrum whey-treated cells. Genes associated with proliferation (thirteen up-regulated genes), adhesion (six up-regulated and one down-regulated genes) and migration (ten up-regulated and one down-regulated genes) were identified. These genes are shown in the volcano plot representing gene expression in colostrum whey-treated cells (Fig. 5). Classification of each up-regulated or down-regulated genes according to biological processes is also presented (Table 3).

**Table 3.** Functional classification (Gene Ontology (GO) biological processes) of genes with increased or decreased expression in colostrum whey-treated IPEC-J2 cells.

Gene symbol	Gene name	Regulation of cell migration (GO:0030334), *P = 1 011 × 10^−7^	Regulation of cell adhesion (GO:0030155), *P = 3·360 × 10^−5^	Regulation of cell proliferation (GO:0042127), *P = 4 707 × 10^−4^	Response to wounding (GO:0009611), *P = 8·904 × 10^−3^
AREG	Amphiregulin			X	
CDKN2B	Cyclin-dependent kinase inhibitor 2B (p15, inhibits CDK4)			X	
CITED2	Cbp/p300-interacting transactivator 2-like	X	X		
CSF2	Colony- stimulating factor 2 (granulocyte–macrophage)			X	
CXCR4	Chemokine (C–X–C motif) receptor 4	X			X
CYP1A1	Cytochrome P450 1A1				X
CYR61	Cysteine-rich, angiogenic inducer, 61	X	X	X	X
F3	Tissue factor	X		X	X
FOS	FBJ murine osteosarcoma viral oncogene homolog				X
GADD45A	Growth arrest and DNA-damage-inducible, alpha				
GADD45G	Growth arrest and DNA-damage-inducible, gamma				
HAS3	Hyaluronan synthase 3				
HBEGF	Heparin-binding EGF-like growth factor	X		X	X
HR	Hairless protein				
IL-8	IL-8	X	X	X	X
NGF	Beta-nerve growth factor-like			X	X
PLAU	Plasminogen activator, urokinase	X	X	X	X
PTGS1	Prostaglandin-endoperoxide synthase 1			X	
RNF39	RING finger protein 39-like				
SERPINE1	Serpin peptidase inhibitor, clade E member 1	X	X	X	X
SLITRK6	SLIT and NTRK-like family, member 6				
SMAD7	SMAD family member 7	X	X		
SOX9	SRY (sex determining region Y)-box 9	X	X	X	
SPB9	Serine protease inhibitor 9				
TAGAP	t-cell activation Rho GTPase-activating protein-like				
TIPARP	TCDD-inducible poly(ADP-ribose) polymerase				
WNT11	Wingless-type MMTV integration site family, member 11	X		X	
XKR8	XK-related protein 8-like				
ZNF503	Zinc finger protein 503-like				

*P values for the Bonferroni test.

## Discussion

The aim of the present study was to survey the potential of colostrum whey to promote intestinal health and to propose cellular mechanisms responsible for these beneficial properties. Therefore, the effects of colostrum whey on biological processes involved in intestinal homoeostasis were compared with the ones observed with cheese whey, a defatted and decaseinated by-product obtained during cheese making. The highest amount of wheys added to cells (10 mg/ml or 1 %) was calculated to match estimated amount of product believed to reach intestinal epithelial cells following ingestion.

We have shown that important biological processes involved in wound healing, including proliferation and migration, were increased by colostrum whey in intestinal epithelial cells. Interestingly, cheese whey failed to induce cell migration, which is a major step in epithelial restitution. These results clearly demonstrated that, although the two products compared in this work had similar ingredients and protein content, fundamental differences between colostrum whey and cheese whey composition could trigger different outcomes on intestinal epithelial cells.

Indeed, amounts of growth factors are generally higher in colostrum compared with milk from late lactation^(^^8^^,^^9^^)^. Since growth factors play important roles in wound healing processes^(^^20^^)^, it is likely that the various growth factors found in colostrum whey are involved in the effects we observed. However, a previous study performed with milk obtained from different stages of lactation also measured variation in growth-promoting activity of human fetal small intestinal cells, and concluded that the concentration level of TGF-β and IGF-I in milk could not fully explain these differences^(^^21^^)^. Also, evidences have shown that milk is significantly superior than EGF, IGF-1, fibroblast growth factor, human growth factor and TGF-α, individually or in combination, to promote growth activity in human intestinal epithelial cells^(^^22^^)^. Lactoferrin, found in colostrum whey, is also known for its beneficial effect on wound healing^(^^23^^)^, yet results obtained in our laboratory showed that lactoferrin is unable to increase cell migration in IPEC-J2 cells (data not shown). Therefore, growth and wound healing–promoting activity of colostrum are complex and most likely multifactorial. Eventually, compositional data on colostrum whey could enable us to further elaborate on the role of each component.

Significant morphological changes were observed when resuspended IPEC-J2 cells were treated with cheese whey before being allowed to attach to culture dish, while changes observed in colostrum whey-treated cells were hardly perceptible. Interestingly, these morphological changes were only observed when cheese or colostrum whey were added to resuspended IPEC-J2 cells, thus unpolarised cells, but no morphological changes were observed when whey products were added to cells already attached to the cell culture dish (data not shown). In Caco-2/15 cells, no morphological change was observed, but a decrease in cell attachment to the culture dish was noticed in colostrum whey-treated cells. While no further investigation was done to determine the cause of the morphological changes observed in IPEC-J2 cells, we believe the induction of an epithelial–mesenchymal transition (EMT) might be involved. In EMT, polarised epithelial cells undergo biochemical changes leading to the acquisition of mesenchymal cell characteristics. These changes include loss of cell adhesion, cell–cell junction dissolution, actin reorganisation, loss of cell polarity, E-cadherin as well as Zona occludens proteins repression and metalloprotease induction^(^^24^^)^. EMT is essential for many biological processes such as wound healing, tissue regeneration and organ fibrosis, but is also associated with initiation of metastasis for cancer progression. It is likely that growth factors present in colostrum and milk, including TGF-β and EGF, as well as their precise proportion play major roles in the induction of these morphological changes, since they are well known for their capacity to promote EMT^(^^25^^–^^28^^)^. Indeed, studies have shown that combination of TGF-β1 with EGF-induced cell migration, invasion and anchorage-independent growth^(^^29^^,^^30^^)^. In fact, in these studies, dramatic morphological changes characteristic of EMT are observed after combination of EGF and TGF-β1 treatment, similarly to the results we obtained following cheese whey treatment.

Finally, results obtained from the microarray analysis revealed that the expression of a significant number of genes involved in biological processes affecting gastrointestinal health was increased or decreased by colostrum whey. Therefore, this suggests that colostrum whey promotes processes involved in wound healing by regulating the expression of a specific set of genes in intestinal epithelial cells. Taken together, our results point out at a distinctive health potential of colostrum-based ingredients, and the next phase should focus on their characterisation.

### Conclusion

In conclusion, we show that a gene expression programme induced by colostrum whey promotes growth and wound healing properties of intestinal epithelial cells. Divergent results obtained following cheese whey treatment indicates that a delicate balance between growth factors and other bioactive components may be crucial for the regulation of intestinal epithelial cell homeostasis. Therefore the unique functionality of colostrum whey in intestinal epithelial cells should be taken into account and investigated further to develop its potential as food additive.

## References

[1] Ferretti G, Bacchetti T, Masciangelo S, (2012) Celiac disease, inflammation and oxidative damage: a nutrigenetic approach. Nutrients 4, 243–257. 22606367 10.3390/nu4040243 PMC3347005

[2] Martínez C, González-Castro A, Vicario M, (2012) Cellular and molecular basis of intestinal barrier dysfunction in the irritable bowel syndrome. Gut Liver 6, 305–315. 22844557 10.5009/gnl.2012.6.3.305 PMC3404166

[3] Iizuka M & Konno S (2011) Wound healing of intestinal epithelial cells. World J Gastroenterol 17, 2161–2171. 21633524 10.3748/wjg.v17.i17.2161 PMC3092866

[4] Efstathiou JA & Pignatelli M (1998) Modulation of epithelial cell adhesion in gastrointestinal homeostasis. Am J Pathol 153, 341–347. 9708793 10.1016/S0002-9440(10)65576-9 PMC1852965

[5] Pignatelli M (1996) Modulation of cell adhesion during epithelial restitution in the gastrointestinal tract. Yale J Biol Med 69, 131–135. 9112744 PMC2588987

[6] Sturm A & Dignass AU (2008) Epithelial restitution and wound healing in inflammatory bowel disease. World J Gastroenterol 14, 348–353. 18200658 10.3748/wjg.14.348 PMC2679124

[7] Guilloteau P, Zabielski R, Hammon HM, (2010) Nutritional programming of gastrointestinal tract development. Is the pig a good model for man? Nutr Res Rev 23, 4–22. 20500926 10.1017/S0954422410000077

[8] Madsen BD, Rasmussen MD, Nielsen MO, (2004) Physical properties of mammary secretions in relation to chemical changes during transition from colostrum to milk. J Dairy Res 71, 263–272. 15354571 10.1017/s0022029904000263

[9] Montoni A, Gauthier SF, Richard C, (2009) Bovine colostrum as substrate for the preparation of growth factor-enriched protein extracts: identifying the optimal collection period during lactation. Dairy Sci Technol 89, 511–518.

[10] Godden S (2008) Colostrum management for dairy calves. Vet Clin North Am Food Anim Prac 24, 19–39. 10.1016/j.cvfa.2007.10.005 PMC7127126 18299030

[11] Vasseur E, Borderas F, Cue RI, (2010) A survey of dairy calf management practices in Canada that affect animal welfare. J Dairy Sci 93, 1307–1315. 20172250 10.3168/jds.2009-2429

[12] Pouliot Y & Gauthier SF (2006) Milk growth factors as health products: some technological aspects. Int Dairy J 16, 1415–1420.

[13] Gauthier SF, Pouliot Y & Maubois JL (2006) Growth factors from bovine milk and colostrum: composition, extraction and biological activities. Lait 86, 99–125.

[14] Brosnahan AJ & Brown DR (2012) Porcine IPEC-J2 intestinal epithelial cells in microbiological investigations. Vet Microbiol 156, 229–237. 22074860 10.1016/j.vetmic.2011.10.017 PMC3289732

[15] Rothkötter HJ (2009) Anatomical particularities of the porcine immune system-a physician's view. Dev Comp Immunol 33, 267–272. 18775744 10.1016/j.dci.2008.06.016

[16] Litten-Brown JC, Corson AM & Clarke L (2010) Porcine models for the metabolic syndrome, digestive and bone disorders: a general overview. Animal 4, 899–920. 22444262 10.1017/S1751731110000200

[17] Patterson JK, Lei XG & Miller DD (2008) The pig as an experimental model for elucidating the mechanisms governing dietary influence on mineral absorption. Exp Biol Med 233, 651–664. 10.3181/0709-MR-262 18408137

[18] Rhoads JM, Chen W, Chu P, (1994) L-glutamine and L-asparagine stimulate Na+ -H+ exchange in porcine jejunal enterocytes. Am J Physiol 266, G828–G838. 8203529 10.1152/ajpgi.1994.266.5.G828

[19] Beaulieu JF & Quaroni A (1991) Clonal analysis of sucrase-isomaltase expression in the human colon adenocarcinoma Caco-2 cells. Biochem J 280, 599–608. 1764023 10.1042/bj2800599 PMC1130497

[20] Barrientos S, Stojadinovic O, Golinko MS, (2008) Growth factors and cytokines in wound healing. Wound Repair Regen 16, 585–601. 19128254 10.1111/j.1524-475X.2008.00410.x

[21] Purup S, Vestergaard M, Pedersen LO, (2007) Biological activity of bovine milk on proliferation of human intestinal cells. J Dairy Res 74, 58–65. 16978432 10.1017/S0022029906002093

[22] Hirai C, Ichiba H, Saito M, (2002) Trophic effect of multiple growth factors in amniotic fluid or human milk on cultured human fetal small intestinal cells. J Pediatr Gastroenterol Nutr 34, 524–528. 12050579 10.1097/00005176-200205000-00010

[23] Takayama Y & Aoki R (2012) Roles of lactoferrin on skin wound healing. Biochem Cell Biol 90, 497–503. 22332789 10.1139/o11-054

[24] Kalluri R & Weinberg RA (2009) The basics of epithelial-mesenchymal transition. J Clin Invest 119, 1420–1428. 19487818 10.1172/JCI39104 PMC2689101

[25] Stoll SW, Rittié L, Johnson JL, (2012) Heparin-binding EGF-like growth factor promotes epithelial-mesenchymal transition in human keratinocytes. J Invest Dermatol 132, 2148–2157. 22592159 10.1038/jid.2012.78 PMC3423535

[26] Moustakas A & Heldin CH (2007) Signaling networks guiding epithelial-mesenchymal transitions during embryogenesis and cancer progression. Cancer Sci 98, 1512–1520. 17645776 10.1111/j.1349-7006.2007.00550.x PMC11158989

[27] Sabe H (2011) Cancer early dissemination: cancerous epithelial-mesenchymal transdifferentiation and transforming growth factor β signalling. J Biochem 149, 633–639. 21478191 10.1093/jb/mvr044

[28] Sipos F & Galamb O (2012) Epithelial-to-mesenchymal and mesenchymal-to-epithelial transitions in the colon. World J Gastroenterol 18, 601–608. 22363130 10.3748/wjg.v18.i7.601 PMC3281216

[29] Uttamsingh S, Bao X, Nguyen KT, (2008) Synergistic effect between EGF and TGF-beta1 in inducing oncogenic properties of intestinal epithelial cells. Oncogene 27, 2626–2634. 17982486 10.1038/sj.onc.1210915

[30] Tian YC, Chen YC, Chang CT, (2007) Epidermal growth factor and transforming growth factor-beta1 enhance HK-2 cell migration through a synergistic increase of matrix metalloproteinase and sustained activation of ERK signaling pathway. Exp Cell Res 313, 2367–2377. 17467690 10.1016/j.yexcr.2007.03.022

